# Towards an integrated blood pressure self-monitoring solution for stroke/TIA in Ireland: a mixed methods feasibility study for the TASMIN5S IRL randomised controlled trial

**DOI:** 10.1186/s40814-023-01240-2

**Published:** 2023-01-13

**Authors:** R. Doogue, P. Hayes, R. Hebert, A. Sheikhi, T. Rai, K. Morton, C. Roman, R. J. McManus, L. G. Glynn

**Affiliations:** 1 School of Medicine, University of Limerick, Limerick, Ireland; 2 Health Research Institute, University of Limerick, Limerick, Ireland; 3 Nuffield Department of Primary Care Health Sciences, University of Oxford, Oxford, UK; 4 Centre for Clinical and Community Applications of Health Psychology, School of Psychology, University of Southampton, Southampton, SO17 1BJ UK; 5 Institute of Biomedical Engineering, Department of Engineering Science, University of Oxford, Oxford, UK; 6 HRB Primary Care Clinical Trial Network Ireland, Galway, Ireland

**Keywords:** Blood pressure, Self-monitoring, Stroke, Feasibility studies, Randomised controlled trial, Qualitative research

## Abstract

**Background:**

Optimising blood pressure (BP) control is one of the most important modifiable risk factors in preventing subsequent stroke where the risk increases by one-third for every 10 mmHg rise in systolic BP. This study evaluated the feasibility and potential effectiveness of blood pressure self-monitoring with planned medication titration, to inform a definitive trial of the intervention, in patients with a previous stroke or transient ischaemic attack (TIA).

**Methods:**

Patients with a history of stroke/TIA and sub-optimal BP control were invited to take part in a mixed methods feasibility study for a randomised controlled trial. Those meeting the inclusion criteria with systolic BP >130 mmHg were randomised to a self-monitoring intervention group or usual care group. The intervention involved self-monitoring BP twice a day for 3 days within a 7-day period, every month, following text message reminders. Treatment escalation, based on a pre-agreed plan by the general practitioner (GP) and patient, was initiated according to the results of these readings. Semi-structured interviews were carried out with patients and clinicians and analysed thematically.

**Results:**

Of those identified, 47% (32/68) attended for assessment. Of those assessed, 15 were eligible for recruitment and were consented and randomised to the intervention or control group on a 2:1 basis. Of those randomised, 93% (14/15) completed the study and there were no adverse events. Systolic BP was lower in the intervention group at 3 months. Participants found the intervention acceptable and easy to use. GPs found it easy to incorporate into their practice activity without increasing workload.

**Conclusions:**

TASMIN5S, an integrated blood pressure self-monitoring intervention in patients with a previous stroke/TIA, is feasible and safe to deliver in primary care. A pre-agreed three-step medication titration plan was easily implemented, increased patient involvement in their care, and had no adverse effects. This feasibility study provides important information to inform a definitive trial to determine the potential effectiveness of the intervention in patients post-stroke or TIA.

**Trial registration:**

ISRCTN57946500. Registered on 12/08/2019.

## Key messages regarding feasibility

- What uncertainties existed regarding feasibility prior to this study?
  TASMIN5S is a multidimensional intervention designed to help lower blood pressure in patients who have had a previous stroke/TIA. Patients are supported to take and record their own blood pressure, with instant feedback of results. A monthly report is sent to their GP. The GP manages the results based on a pre-agreed treatment plan. Prior to testing the effectiveness of this multi-component intervention we wanted to explore the feasibility and acceptability of implementing this intervention with both patients and GPs, test the randomisation and data collection methods and get feedback from patients and GPs that would inform necessary changes to the design for a full randomised controlled trial (RCT).
- What are the key feasibility findings?
  Patients found the intervention acceptable and easy to use. The majority (>90%) completed the 3-month trial and indicated willingness to continue with the intervention. The electronic messaging and feedback system worked without any failures. Valuable information on invitation and recruitment processes was obtained. GPs found the intervention acceptable and easy to implement without adding to their workload. More efficient methods of receiving results in the practice were identified for further trials.
- What are the implications of the feasibility findings for the design of the main study?
  The findings from this study provided information on the recruitment process and the importance of GP support, and a better understanding of the number of practices that will need to be recruited for a full trial. Many of the volunteers when assessed were excluded as their BP was well controlled. This is important information for recruitment planning for a full trial. We identified improvements to the results delivery system which will make the intervention more efficient for the GP. All participants in this trial preferred the option of free text messaging to record their BP results. However, having the options of an App and website will continue to be available for patients who might prefer these options in the full trial.

## Background

Stroke is one of the leading causes of death and disability worldwide. Despite improvements in the prevention and management of stroke, the incidence of stroke continues to grow especially with an ageing population [1]. The economic burden of stroke in Europe is significant, increasing from €45 billion per year in 2015 [2], to more recent estimated costs of €60 billion per year [3]. The individual effects of stroke are even more significant with many people leaving the hospital after a stroke suffering from limb impairment or aphasia (impacting the ability to read, write or understand language), often resulting in devastating personal, social or economic consequences.

We acknowledge that primary prevention of stroke/TIA (hereafter referred to as stroke) must be our first aim, in order to prevent these debilitating consequences. However, in those who have had a previous stroke, it is vital that we focus on risk factor modification to prevent further events. Subsequent stroke has an even higher incidence of disability and death, with cumulative risks of further stroke increasing from 3% after 1 month to 40% 10 years after the first stroke [4]. Secondary prevention has the potential to reduce stroke reoccurrence by up to 80%. Measures include smoking cessation, reducing salt intake, moderate alcohol intake, achieving a healthy BMI, increased exercise, dietary modifications with increased fruit and vegetable intake and controlling blood pressure to a target of <130/80 mmHg if tolerated [5]. Of these, high blood pressure has been shown to be one of the most important modifiable risk factors in the secondary prevention of stroke, with evidence suggesting that lowering systolic BP by 10 mmHg can reduce the incidence of stroke by one third and that further reduction in BP where tolerated can have an even more beneficial effect, regardless of the blood pressure level before treatment [6].

Controlling blood pressure in this complex group of patients can be challenging. A survey of secondary prevention of stroke in Europe has shown that satisfactory levels of blood pressure control were found in less than 60% of countries [7]. A recent cross-sectional survey in Irish General Practice [8] demonstrated that one-third of patients did not have their blood pressure adequately controlled. The reasons for sub-optimal BP control are multi-faceted. These include patient factors where adherence may be an issue [9, 10] and physician factors, which may also include therapeutic inertia [10], lifestyle issues and treatment-resistant hypertension [11].

Developing novel strategies to optimise BP control in this group of patients could have potential benefits in reducing the incidence of further stroke. GP-supervised self-monitoring and self-management of blood pressure have been shown to be effective in improving blood pressure in primary care [12, 13]. Due to the often-debilitating outcomes in patients following stroke, it is important to investigate if a self-monitoring intervention can be used by this patient group and if it is effective.

## Aims

This study aimed to provide evidence that would inform a definitive RCT of an intervention involving blood pressure self-monitoring, with planned medication titration, among patients with a previous stroke/TIA. It will evaluate the feasibility of recruitment and retention to the trial, the acceptability of the intervention among participants and clinicians and the potential effectiveness of the intervention.

## Methods

### Trial registration and ethics approval

This feasibility study was adapted from the TASMIN5S protocol (ISRCTN57946500), a multi-centre RCT examining the effects of blood pressure self-monitoring in patients with a previous stroke or TIA which took place concurrently in the UK. Research ethics approval was received from University Hospital Limerick Research Ethics Committee (Ref: 077/19). We have followed CONSORT guidelines for the reporting of feasibility studies [14].

### Study design

The study design was a prospective two-group, unmasked feasibility study for a randomised controlled trial. Participants were allocated to the intervention or control group on a 2:1 basis. An internet-based randomisation process was used. The trial was carried out in the mid-west of Ireland.

### Patient and public involvement

Stroke patients and advocates from the Stroke Association (www.stroke.org.uk) were included on the advisory committee overseeing the development of the main intervention. Community groups such as Different Strokes Southampton and the Oxford Aphasia Group were asked for feedback during the intervention development.

The intervention development process involved stroke patients from both Ireland and the UK providing intervention and material suitable for diverse groups of people from both Irish and UK health care environments. This helped to identify potential barriers and improve the accessibility of the intervention [15], resulting in patient material that was easy to read, aphasia friendly and suitable for people with lower literacy levels.

### Participants

General practices in counties Limerick and Clare, in the mid-west of Ireland, who had taken part in a recent cross-sectional survey of stroke and TIA [8] were invited to take part in the feasibility study. All practices used electronic medical records (EMRs) and cared for both public (funded through the Primary Care Reimbursement Service (PCRS) scheme [16]) and private (fee-paying) patients. Adults over 18 years, with a history of a previous stroke or TIA, with a recent office blood pressure >130 mmHg systolic, who met the inclusion criteria (Appendix 1), were identified by their GP from their practice EMRs and invited to participate.

### Recruitment

The study commenced in October 2019. Study information was sent to each participating GP, which included study eligibility (Appendix 1) and exclusion (Appendix 2) criteria, a short online information programme for health care professionals and a copy of all study materials. GPs who were willing to participate in the study were asked to send an invitation letter and short study information leaflet (Appendix 3) to identified patients, outlining the proposed feasibility study and inviting them to take part. The patient information leaflet providing details of the study was in pictorial format to allow better understanding for those with possible aphasia post-stroke or those with lower literacy levels [15]. Patients were invited to return the reply slip giving their permission to be contacted by the researcher in order to receive further information about the study and make an appointment for study eligibility assessment. Patients who did not want to take part could return the reply slip indicating same and a reason why not. Non-responders were followed up by the GP 2–3 weeks later by phone and provided with further information. They were invited to return the reply slip if interested.

Two practices were recruited prior to the Covid-19 lockdown in Ireland (December 2019–February 2020). Participants, who had given their permission, were contacted by the researcher and invited to attend an assessment appointment in their own GP practice. Following informed consent and assessment, eligible patients with BP >130 mmHg (average of second and third reading after sitting for 5 min, using the BP-TRU monitor [17]) were randomised to the intervention (self-monitoring) or control (usual care) group using an Internet-based randomisation key [18].

A further two practices were recruited during the Covid-19 lockdown (August 2020–September 2020) following ethical approval for an amendment to the protocol allowing for the recruitment of patients to the study remotely. Participants were assessed by phone or video consultation after receiving all study materials by post. The amended protocol exactly mirrored the original protocol except for all interactions with the patient took place virtually and the patient measured their BP at home under the instruction of the researcher by video link.

Baseline data collected during the assessment included patient characteristics and demographics, level of education, past medical history including comorbidities, number of antihypertensive medications currently prescribed, short orientation memory concentration test [19] and blood pressure readings including an assessment of orthostatic hypotension.

### Randomisation

Internet-based randomisation with phone backup was used [18]. The randomisation key was held by an independent researcher not involved in the recruitment or the assessment of participants. When a participant was eligible for recruitment, the independent researcher was contacted by text message and the allocation group was advised. As the main objective of this study was to explore feasibility, randomisation was on a 2:1 intervention to control basis.

### Intervention

The TASMIN5S intervention used a digital telemonitoring platform called BP: Together (established during the intervention development phase [15]), which was set up to allow the sending and receiving of free text messages to mobile phones. Results of the patients’ home BP readings were automatically collated from the digital platform and sent to each patient’s individual GP by email.

Participating GPs completed a 30-min online training programme before the study commenced. It outlined the objectives of the study, provided a rationale for lowering stroke patients’ blood pressure to the intervention targets (<125 mmHg systolic on home readings), gave practical information on how they would receive the patient’s home BP readings, provided the GP with a review of current guidelines for BP control in Stroke or TIA [11, 20] and provided suggestions on BP management strategies including pharmacological intensification [12].

The self-monitoring intervention group received a BP monitor (Omron M10-IT [21]) and were instructed on how to take their own BP and how to send the BP readings using the digital platform of choice:

1. Free text message to the BP: Together platform
2. Via the BP: Together App downloaded to their phone
3. Via the BP: Together website

Pre Covid-19, patients received instruction from the research nurse following assessment and randomisation in their GP surgery, with a take-home instruction booklet provided. During the Covid-19 lockdown, patients received the same instruction from the research nurse by phone/video consultation, with the instruction booklet provided by post with the BP monitor.

Following the baseline assessment, GPs were sent an email with a list of their patients randomised to the intervention (self-monitoring) and control (usual care) groups. Patients in both groups received a medication review by their GP. During this baseline medication review, patients in the intervention group were given an individualised three-step treatment escalation plan. This plan would be enacted by the GP if there was a clinical indication, based on the results of the home BP readings received by email following each monitoring period. The control group continued with usual care for the duration of the feasibility study.

**Fig. 1:**
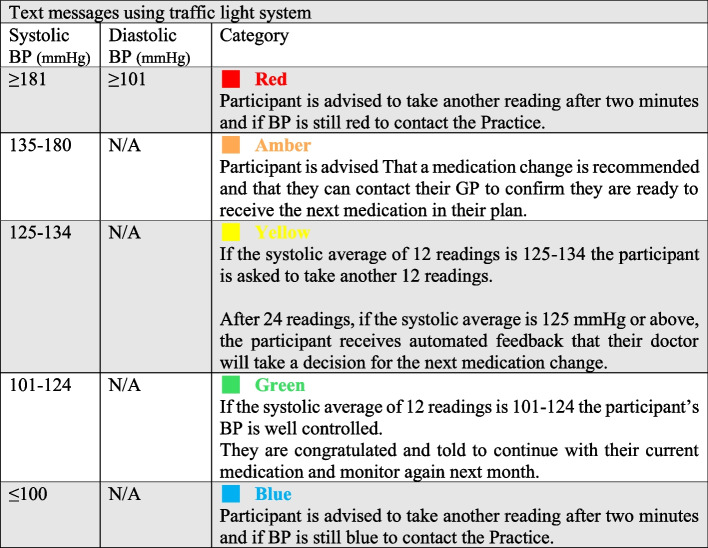
Text messages using traffic light system

Self-monitoring involved recording BP twice a day for 3 days within a 7-day period, every month, following text message reminders. Patients sent their BP readings by free text to the BP: Together digital platform. The monthly average BP was sent to the patient using a traffic light system (Fig. 1) and to the patient’s GP immediately after each monitoring period. Treatment escalation was initiated by the GP if indicated, according to the three-step medication plan (Appendix 4). The feasibility study lasted 3 months.

### Post-study interviews

All patients randomised to the intervention arm and all participating GPs were invited to take part in a post-study interview. We developed two semi-structured interview guides—one for patients and one for GPs—in order to obtain their respective feedback about the intervention. The interview guides were developed using a planned approach which identified prerequisites and current knowledge, to encourage patients and the GPs to provide feedback on the intervention [22].

### Outcomes

The primary outcomes of this study were

1. Feasibility of recruitment of practices and participants:

- The proportion of GP practices willing to participate in the study and the retention rate
- The proportion of patients eligible for screening, assessment and randomisation
- The proportion of patients consenting to the intervention and completing the trial

2) Acceptability of the intervention:

- The proportion of GP practices and patients completing the study
- Acceptability of the intervention, including adherence to the intervention and likelihood of recommending the programme to other stroke patients, the number of adverse events recorded and the effects of the intervention on GP time and workload, measured using thematic analysis of post-trial interviews

3) Change in systolic and diastolic blood pressure from baseline to end of the trial

Secondary outcomes included the delivery of the intervention, the number of blood pressure readings taken and the number of text messages successfully sent and received between the patient and the BP: Together platform.

### Sample size

As this was a feasibility study, there were no formal power calculations before commencing the study [23].

### Data analysis

Quantitative data analysis was carried out using SPSS [24]. Descriptive statistics were used to compare the participants’ baseline characteristics. Continuous variables are reported as mean (± standard deviation (SD)) and categorical variables are presented as count (percentage). Formal tests of statistical significance were not carried out, as this study was not conducted or powered to detect effects on BP.

Thematic analysis was used to explore the emerging themes from the post-trial interviews with both patients and participating GPs. The five stages of the Framework Process were followed in the examination of the qualitative data which included familiarisation, thematic framework identification, indexing, charting, mapping and interpretation [25]. Coding was partially conducted with another researcher from a different professional background for inter-coder reliability [26]. To heighten reflexivity, three members of the research team (a research nurse and two general practitioners) reviewed all the data and contributed to the thematic analysis [27]. QSR International NVivo 12 software [28] was used to organise and code the transcripts to facilitate the analysis and comparison of relationships between the coded ideas [29].

## Results

Eight out of the ten practices invited to take part in the study indicated willingness to participate when contacted initially. Due to the effects of Covid-19, four of these practices withdrew, stating that they did not have the capacity to participate due to the increased workload associated with the Covid-19 pandemic but would have taken part otherwise. In total, four of the ten general practices invited were recruited.

The four practices that participated were representative of Irish general practice [30] in size and type, teaching and non-teaching. They consisted of a large urban, small urban, large mixed urban and rural, and small rural practice. The initial assessment for trial eligibility, following a computer search of the practice EMRs, identified 233 patients with a previous stroke or TIA. Of these, there were 95 potential patients who met the inclusion criteria (Appendix 1) and were eligible for invitation to the study. Following review by the GP, 27 patients were excluded for the following reasons: BP self-monitoring was not suitable for patients, patients had left practice, patients had a diagnosis of dementia which had not been coded correctly and patient was deceased. This resulted in 68 patients eligible for invitation.

Thirty-seven of the patients identified (54.4%) agreed to attend for assessment. Of these, 18 (26.5%) agreed to take part after the mailout and a further 19 (27.9%) agreed to take part following a call from the GP. During the follow-up phone call by the GP to non-responders, practical barriers that were identified included concerns about being able to take their own BP and send the BP results as required. The follow-up phone call from the GP reassured patients and doubled participation in the study.

**Fig. 2:**
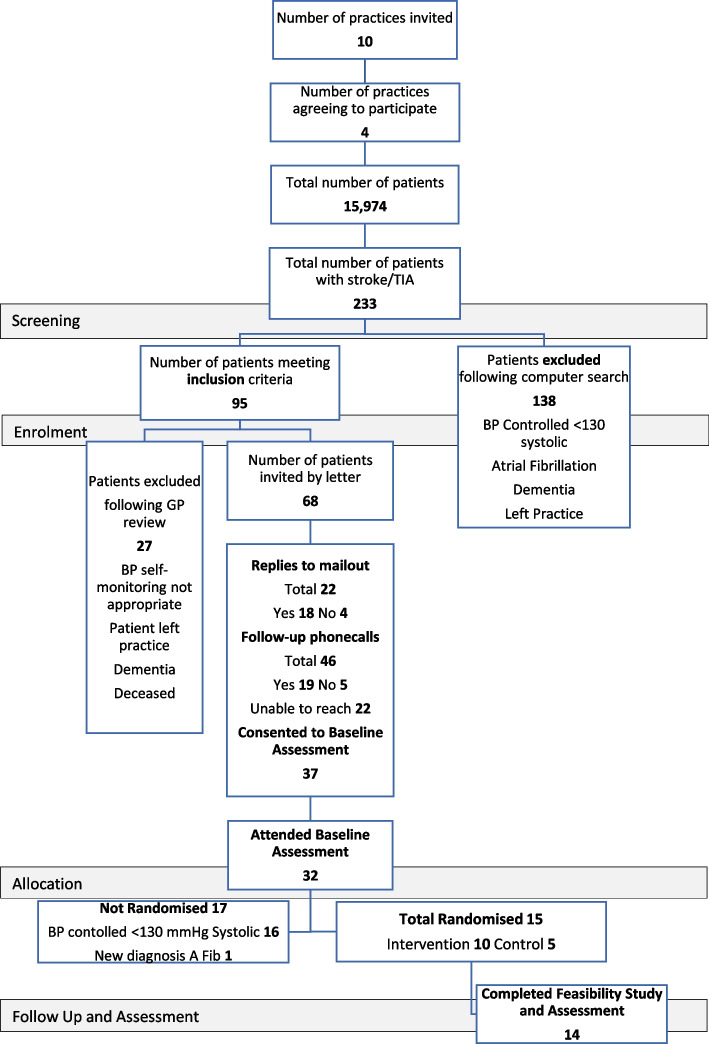
CONSORT diagram showing flow-through study

Thirty-two of the 37 volunteers attended baseline assessment. Of these, 15 were eligible for randomisation. Seventeen patients were not eligible to take part, 16 had BP controlled below 130 mmHg systolic and one person had a new diagnosis of atrial fibrillation. See CONSORT diagram for further details (Fig. 2). The 15 eligible patients were randomised on a 2:1, intervention (self-monitoring) to control (usual care) basis.

Participant characteristics are described in Table 1. These are presented as total participants, intervention and control groups. Baseline characteristics were similar in both study arms. The mean age of the total group was 70 years. Eighty percent were male and 53.3% lived alone. Almost three-quarters (73.3%) of participants had suffered a previous stroke while the remaining quarter (26.7%) had a TIA. All levels of education were represented, including those who had a primary education only, to postgraduate levels of education.

**Table 1:** Baseline characteristics

Participant characteristics	Intervention Self-monitoring ***N*** = 10	Control Usual care ***N*** = 5	Total ***N*** = 15
**Demographics**			
Age, mean (SD)	69 (7.1)	71.2 (8.7)	70 (7.4)
Male, *n* (%)	8 (80%)	4 (80%)	12 (80%)
Lives alone, *n* (%)	6 (60%)	2 (40%)	8 (53.3%)
Helped by carer, *n* (%)	3 (30%)	1 (20%)	4 (26.7%)
Stroke, *n* (%)	7 (70%)	4 (80%)	11 (73.3%)
TIA, *n* (%)	3 (30%)	1 (20%)	4 (26.7%)
**Education (highest level achieved)**			
Primary education only, *n* (%)	2 (20%)	1 (20%)	3 (20%)
Secondary school certificate, *n* (%)	3 (30%)	2 (40%)	5 (33.3%)
University degree or higher, *n* (%)	4 (40%)	2 (40%)	6 (40%)
Diploma/other qualification, *n* (%)	1 (10%)	0	1 (6.7%)
**Comorbidities**^a^			
Myocardial infarction, *n* (%)	0	1 (20%)	1 (6.7%)
Angina, *n* (%)	0	1 (20%)	1 (6.7%)
CABG/angioplasty (balloon)/stent, *n* (%)	3 (30%)	1 (20%)	4 (26.7%)
Peripheral Vascular Disease, *n* (%)	0	0	0
Diabetes—type 1, *n* (%)	0	0	0
Diabetes—type 2, *n* (%)	1 (10%)	1 (20%)	2 (13.3%)
Heart failure, *n* (%)	0	0	0
**Current antihypertensive medication**			
0, *n* (%)	0	0	0
1, *n* (%)	4 (40%)	1 (20%)	5 (33.3%)
2, *n* (%)	6 (60%)	4 (80%)	10 (66.7%)
Range	1–2	1–2	1–2

^a^Two patients had more than one comorbidity

The mean systolic BP at baseline was 143.7 mmHg in the intervention group and 146.2mmHg in the control group and the mean diastolic BP was 79.3 mmHg and 78.6 mmHg respectively (mean of second and third readings after sitting for 5 min) (Table 2).

**Table 2:** BP readings pre and post-feasibility study

	Intervention Self-monitoring (***n*** = 9)	Control Usual care (***n*** = 5)
**Baseline BP (mean of 2nd and 3rd readings)**		
Systolic BP, mean (SD)	143.7 (9.49)	146.2 (9.42)
Diastolic BP, mean (SD)	79.3 (11.37)	78.6 (15.14)
**Post-trial BP**^**a**^ **(mean of 2nd and 3rd readings)**		
Systolic BP, mean (SD)	120.8 (16.35)	140.5 (3.86)
Diastolic BP, mean (SD)	69.4 (9.07)	73.1 (14.97)

^a^The change in BP between baseline and post-trial cannot be compared for statistical significance due to the small numbers in the feasibility study

The 3-month feasibility study was completed by 14 out of the 15 participants. The participants who completed the trial adhered to the protocol of monthly BP measurement and recording for 3 months, sent their readings to the trial platform and agreed to treatment adjustment with their GP. One participant dropped out of the trial after 1 month reporting to the GP that it was too time-consuming. All participants chose to text their blood pressure results rather than using the BP: Together App or website. They found text messaging simple to use and preferred it as many had concerns over unreliable internet access. Two participants received help from a spouse or carer. One needed help to send the results by text message. The other needed help to take the BP readings and send the results. All participants were given a contact number for the research nurse if they had any questions relating to the procedure for taking their BP, recording the results or texting their results. There were no differences in intervention adherence or trial completion between the group assessed and taught how to use the intervention by the research nurse in surgery versus the group at home.

All post-trial BP readings were obtained by video-link with the research nurse instructing the patient using the exact same protocol as used in the baseline assessment. Blood pressure dropped in both intervention and control groups. The drop in both the systolic and diastolic BP readings was higher in the intervention group than that in the control group. Systolic BP dropped from 143.7 mmHg at baseline to 120.8 mmHg post-trial in the intervention group and from 146.2 to 140.5 mmHg in the control group. Diastolic BP dropped from 79.3 to 69.4 mmHg in the intervention group and from 78.6 to 73.1 mmHg in the control group. However, these cannot be compared for significance due to the small numbers involved, and the primary aim of this study was to test the feasibility of the intervention (Table 2).

### Post-trial interviews

All participants who completed the intervention and all GPs consented to a post-pilot trial interview. Following thematic analysis, emerging themes from patient interviews included perceived benefits or negative effects of blood pressure self-monitoring and the prescribed intervention, willingness to continue with the intervention and likelihood of recommending the programme to other survivors of stroke. The main themes emerging from the GP interviews were as follows: the benefits of BP self-monitoring, managing the intervention and incorporating it into practice systems, and the potential impact on their workload.

### Participant views

All nine participants who completed the intervention took part in a structured phone interview. The majority of these found the intervention easy to use, acceptable and did not cause any anxieties or concerns but on the contrary appeared to reduce anxiety around blood pressure. Pseudonyms are used in place of participant’s names.

I think it’s great that you can self-monitor and get a better feeling for your own health. (Mark, 69 years)

I found it very helpful and I relaxed a bit more about my blood pressure knowing that it was being monitored by the doctor. (Linda, 71 years)

However, one participant found it caused her to feel anxious even though she would not normally class herself as being an anxious person. She was very pleased when the 3-month pilot was over and stated that she would be reluctant to continue in a full 12-month trial.

…it seemed to be very high (BP) and I don’t know. In the end I was kind of dreading it. (Kathleen, 73 years)

This highlights the importance of ensuring patients can access support if they are feeling anxious, especially if their BP readings are high. Support from her GP or the research team may reduce her anxiety and give her the confidence to continue with the intervention.

Some indicated that it motivated them to adhere to a healthier lifestyle including an improved diet and increased exercise.

Because when my blood pressure was up, I was cutting back on a couple of things. I was cutting back on salt and butter and this and that so I think it did help me. (Jack, 57 years)

Many of the participants stated that it encouraged them to become more involved in their care. They discussed their readings with the GP and the possible need for a medication change.

When we were checking over a few weeks or a month or a two-month period, it was obvious that my medication had to be increased, which helped as well, which might never had happened, you know, if I didn’t have it. (Mark, 69 years)

I was in with my GP and I said to him about a change of tablets. (Peter, 68 years)

…very helpful, and the fact last time I was with the GP he changed my medication. (John, 82 years)

When asked, participants stated that they would be happy to participate in a similar project or full 12-month trial, and some felt it would be something they would like to have as part of their care package on an ongoing basis.

I’d be quite happy to do it if it was of benefit to him (GP) or to myself. (Robert, 62 years)

Certainly, I would recommend it, anybody of my age I would recommend to have a monitor. (John, 82 years)

In general, patients found the intervention acceptable and easy to use. However, this intervention may not suit all patients post-stroke. It is important that they understand that they can contact their GP for reassurance if their readings are high and that they have clear information on how to contact the research nurse for further information or to answer any questions. Results from this qualitative work have demonstrated key outcome measures of recruitment, retention, adherence to the protocol and general acceptability of the intervention.

### GP views

Individual semi-structured interviews were carried out with the four participating GPs. They found the intervention acceptable and easy to incorporate into their practice activity. They did not identify an increased workload as the general consensus was that this was a clinical activity that needed to be done anyway.

…because you know it’s something that has to be done, it would have a negligible impact really. We set aside time for checking results and acting on them anyway. (GP, small urban practice)

Getting regular updates and information about somebody’s blood pressure control, has the potential to, I’ve no doubt, save consultations on the one hand, and certainly save in terms of morbidity and mortality associated with potential events that you're preventing. (GP, small rural practice)

There was a general recommendation for results to be delivered via their standard practice electronic results delivery system Healthlink (the national hospital and labs results system) rather than by email, which would ensure that the results were reviewed and actioned during practice hours and in line with standard practice protocols for reviewing results.

It would be preferable if it could be linked directly to the EMR the electronic patient medical record. (GP, small rural practice)

If it was connected with the GP software that would be more user friendly. (GP, large mixed urban and rural practice)

They had no problems making treatment decisions based on the results of the home BP readings and commented that it encouraged their patients to become more involved in their care.

…people are living much longer lives, they are living with multiple chronic diseases, so I guess making patients not just passive receivers of a service or just passive recipients of care, but rather, making them sort of active agents in their own health and wellness, I think is really important. I think technology and this example of technology is a great enabler in terms of that. (GP, small rural practice)

From what I can see, yeah, delighted with it and delighted to be involved in their care. (GP, large urban practice)

They all indicated a willingness to continue with the intervention for a full trial or as part of a patient’s ongoing care plan and felt that patients were happy with the system.

I was amazed about some of my patients, I was worried that some of them might not be able to, you know, or might have reservations about partaking, but I mean they all seem to like it. (GP, small urban practice)

## Discussion

### Summary

We have demonstrated that blood pressure self-monitoring using the TASMIN5S system of digital feedback is both feasible and acceptable with stroke patients and GPs. This study reports on the use of a GP-supervised self-monitoring system for patients, with agreed treatment escalation following the feedback of results via a digital platform. GPs were willing to take part in the study with eight out of ten practices consenting to take part when approached initially. There was no reported effect on practice time or increase in GP workload. Of the patients randomised, 93% (14/15) completed the study and there were no adverse events. A significant reduction in systolic BP and diastolic BP was seen in the intervention group at 12 weeks.

### Comparison with existing literature

The current ESC/ESH (European Society of Cardiology/European Society of Hypertension) guidelines recommend a target systolic BP of 120–130 mmHg [11] for patients post-stroke. There is strong evidence that lowering blood pressure to optimal targets has a positive effect on the prevention of further stroke [31, 32]. Our study aimed for a systolic BP target of less than 125 mm Hg on home readings. Many participants reached this target BP without any adverse effects.

Current hypertension guidelines encourage the use of home monitoring especially in patients with a high cardiovascular risk [33], although some patients owned a blood pressure monitor, none of them had a systematic plan of self-monitoring or knew when to feedback the results to their GP.

There is an increasing body of literature examining the potential beneficial effects of blood pressure self-monitoring, particularly when associated with co-interventions [34–37]. Although improvements in BP control were often small in these studies, this may still have an important clinical effect in reducing vascular complications in hypertensive patients. Self-monitoring empowers patients, is cost-effective and is well-tolerated [38]. It has been shown to be superior to office BP monitoring in predicting end-organ damage [39]. Combining blood pressure self-monitoring with co-interventions such as telemonitoring and tailored support for patients may increase these benefits [35]. Designing co-interventions which involve text messaging systems as used in our study was found to be convenient, accessible and easy to manage. None of our participants chose the option of web-based systems, all of them citing concern re Internet coverage. Many were living in rural areas and were more confident in sending and receiving text messages.

Results from this study are similar to other recent studies showing that high-risk individuals with significant cardiovascular comorbidities are able to self-monitor and titrate medication adjustments in conjunction with their GP [12]. Electronic messaging is increasingly being used as a support mechanism in person-centred goal achievement and has been found to be acceptable by patients in the secondary prevention of stroke [40] which is also clear in the qualitative data and the completion rate seen in our study.

### Strengths and limitations

The strengths of this study include the combination of quantitative outcomes and qualitative data, to develop an in-depth understanding of the feasibility and acceptability of this intervention. It demonstrates the implementation of the intervention in varied contexts, both before and during the Covid-19 pandemic.

Limitations to this study include the small number of general practices participating in the study due to the effects of the Covid-19 pandemic, resulting in fewer patients recruited to the study than originally planned. The original aim was to recruit 50 patients for this feasibility study which is consistent with recommendations in the literature and other similar studies in this field [41–43]. However, despite smaller numbers than planned, this research provides key feasibility findings which will help in the design and development of a definitive randomised controlled trial. It should also be noted that participants were only asked to self-monitor for 3 months, so the findings regarding levels of engagement and acceptability may not apply when people are asked to self-monitor for a longer period of time. For example, it is not known how the anxiety brought about in one participant by ongoing high readings might impact her longer-term engagement with the intervention.

### Implications for research and practice

The current study has demonstrated successful recruitment and retention of patients in an intervention using blood pressure self-monitoring in patients post-stroke. The intervention was acceptable to GPs without increasing their workload and improvements were identified that would allow results to be incorporated more easily into their current practice-based systems. Knowledge gathered on the number of eligible patients post-stroke/TIA in each practice, invitation response rate, exclusion rate and retention rate will help to inform power calculations and determine how many practices would need to be recruited for a definitive trial. This information will ensure a high-quality, well-designed future trial essential for assessing the efficacy of the intervention.

This study demonstrated a preference for the use of text messaging for delivery and feedback of results, especially among older patients and those where broadband coverage may be an issue such as in rural regions. Recruitment of a more diverse group of patients was improved following a phone call from the GP offering support and encouragement to the patient. The intervention was easily managed by patients with aphasia or lower literacy levels with aphasia-friendly literature provided to all participants, and where necessary, support from a carer or family member was encouraged. System improvements for the transfer of results to GPs were identified. Patient and Public Involvement (PPI) informed the various iterations of the research design process, providing an intervention that was accessible and acceptable to both patients and clinicians.

Although the initial study protocol did not plan to recruit and assess patients in their homes, the advent of Covid-19 challenged the researchers to make changes to the original protocol in order to allow the study to continue. The results of this were striking with an equal number of patients consenting to participate virtually as did face-to-face. There was no difference in the completion rates for the virtual versus face-to-face groups. This provides important information for future research where virtual recruitment should be considered as an option for recruitment to future trials.

## Conclusion

Blood pressure self-monitoring, using an integrated feedback system, in high-risk cardiovascular patients post-stroke or TIA is both feasible and acceptable. The use of a pre-agreed three-step medication titration plan was easily implemented, increased patient involvement in their care, improved BP control and had no adverse effects. The results of this feasibility study provide valuable information for the development of a definitive RCT to determine the potential effectiveness of this intervention in patients post-stroke or TIA.

## Data Availability

Data from this clinical trial will be available upon reasonable request to the corresponding author.

## Appendix 1

### Participant inclusion criteria

1. Participant is willing and able to give informed consent for participation in the study

2. Male or female, aged 18 years or above

3. Previous history of stroke and/or specialist confirmed TIA at least 1 month before randomisation

4. Baseline systolic blood pressure > 130 mmHg (mean of 2nd and 3rd readings after 5-min sitting)

5. Willing and able to comply with all study procedures

## Appendix 2

### Participant exclusion criteria

1. Diagnosis of dementia

2. Score over 10 on the short orientation memory concentration test

3. Any other significant disease or disorder which, in the opinion of the Investigator, may put the participants at risk because of participation in the trial

4. Pregnancy

5. BP > 180/110 mmHg at baseline (to be referred to GP but could be subsequently rescreened if BP < 180/110 mmHg)

6. Patient taking more than three antihypertensives

7. Unwilling to self-monitor

8. Receiving care for blood pressure by a specialist rather than a primary care physician

9. Stage 4 CKD or worse, i.e. eGFR less than 30 mL/min/1.73 m2 (as these persons are more likely to be managed by specialists, are more prone to acute kidney injury and/or hyperkalaemia and are less responsive to certain antihypertensives)

10. Diagnosed atrial fibrillation

11. Orthostatic hypotension: more than 20mmHg systolic drop after standing for 1 min. Not relevant if the participant is unable to stand

12. Participation in other studies that concern hypertension and/or adjustment of antihypertensive medication

## Abbreviations

TIA: Transient ischaemic attack
IRL: Ireland
UK: United Kingdom
BP: Blood pressure
GP: General practitioner
RCT: Randomised controlled trial
BMI: Body mass index
EMRs: Electronic medical records
PCRS: Primary Care Reimbursement Service
SD: Standard deviation
ESC/ESH: European Society of Cardiology/European Society of Hypertension
PPI: Patient and Public Involvement
